# Smart Maintenance Solutions: AR- and VR-Enhanced Digital Twin Powered by FIWARE

**DOI:** 10.3390/s25030845

**Published:** 2025-01-30

**Authors:** André Costa, João Miranda, Duarte Dias, Nuno Dinis, Luís Romero, Pedro Miguel Faria

**Affiliations:** 1 IPVC—Instituto Politécnico de Viana do Castelo, 4900-347 Viana do Castelo, Portugal; afilipecosta@ipvc.pt (A.C.); joao.miranda@ipvc.pt (J.M.); duartedias@ipvc.pt (D.D.); romero@estg.ipvc.pt (L.R.); 2 RIOPELE, 4770-405 Vila Nova de Famalicão, Portugal; nuno.dinis@riopele.pt

**Keywords:** internet of things, data analysis, augmented reality, virtual reality, predictive maintenance, industrial digitization, system integration, operational efficiency

## Abstract

In the modern era of industrial digitalization, the convergence of the Internet of Things (IoT), advanced data analysis, augmented reality (AR) and virtual reality (VR) is significantly transforming various industrial sectors. This research aimed to study and develop a proposal for an integrated system that combines IoT, data analysis, AR and VR for the monitoring and maintenance of industrial equipment. The importance of this research lies in its potential to contribute to the implementation of predictive maintenance solutions, which can significantly reduce machine downtime in an industrial environment and thus reduce or prevent operational failures. The central research question of this work was the following: how can the integration of IoT, data analysis and augmented and virtual reality contribute to optimizing industrial maintenance? We tested the combination of technologies to enable the creation of an effective predictive maintenance system, capable of alerting operators to anomalous conditions and providing detailed visual instructions for maintenance tasks. As a result, a prototype system was developed and tested, and it has shown the potential to evolve into a real system in an industrial environment.

## 1. Introduction

In the modern era of industrial digitalization, technologies such as the Internet of Things (IoT), FIWARE, visualization platforms like Grafana, advanced databases, APIs, artificial intelligence (AI), augmented reality (AR) and virtual reality (VR) are profoundly transforming various industrial sectors. These technologies have been extensively studied for their ability to optimize industrial processes and reduce operational costs, driving the progress of Industry 4.0. Recent studies emphasize the relevance of these approaches. For instance, Ref. [1] explored a framework based on a digital twin for predictive maintenance in industrial environments, highlighting the integration between physical systems and their digital twins to enhance the maintenance efficiency and improve the failure prediction accuracy.

Digital twins (DTs) have emerged as a key solution in Industry 4.0, offering real-time simulations and performance predictions for physical systems. Ref. [2] discusses how DTs can integrate operational, environmental and sensor data to build robust predictive models, minimizing unplanned equipment and machinery downtime, thereby boosting business competitiveness. Technologies associated with the Internet of Things (IoT) and machine learning provide unique opportunities for the real-time monitoring and efficient management of industrial assets. A recent study [3] highlighted that manual updates performed by process specialists are effective in monitoring deviations between DTs and physical systems. However, the same study notes that continuous real-time updates can present limitations, such as inadvertently altering critical parameters, which may compromise the predictive accuracy of the models.

The authors of [4] explored the role of DTs and IoT in unlocking operational intelligence, emphasizing their impact across various sectors beyond manufacturing, such as healthcare, transportation and agriculture. This concept of cross-sectoral application underscores the transformative potential of DTs, enabling the integrated and predictive management of industrial resources.

Technologies like virtual reality, according to [5], have enabled effective training in equipment maintenance, allowing participants to enhance their skills and continue learning. VR also offers the advantage of being applied in risk management, helping to identify hazards before they become problems. However, validating the compatibility of VR applications remains a challenge due to the wide variety of existing devices.

Additionally, the application of augmented reality (AR) systems in maintenance contexts has proven beneficial, as demonstrated by [6] in their latest study. Their research presented these systems as effective solutions to support less experienced workers in maintenance tasks, facilitating remote diagnostics and repairs with the assistance of experts and 3D models. The utility of these approaches has been validated by positive feedback from various technicians and supervisors.

In this context, our work proposes an innovative solution that combines these technologies into an integrated system tailored to the needs of predictive maintenance in industrial environments. This system stands out by jointly applying IoT for real-time data collection, FIWARE to ensure system interoperability, Grafana for efficient data visualization, AI for predictive analysis and AR/VR to provide immersive visual guidance and training for machine operators.

This system aims to enhance the optimization of machine predictive maintenance in shop floor environments, reducing downtime while increasing the safety and operational efficiency.

The central question of this research is the following: how can the integration of emerging technologies optimize industrial maintenance while offering greater safety and efficiency? Studies such as [7] highlight the challenges in implementing such solutions, particularly the use of AI and ontologies to predict real-time failures. However, they also underline the opportunities for significant advancements in predictive asset management.

The hypothesis guiding this work is that integrating these technologies will create a system capable of not only identifying anomalous conditions but also providing detailed visual and auditory guidance for operational interventions in industrial machinery. This model will contribute to a more efficient and proactive approach to industrial asset management, aligned with the needs of Industry 4.0.

The article is organized into several sections that address different aspects of the development and implementation of a FIWARE-based digital twin supported by augmented reality (AR) for predictive maintenance. The structure follows a logical approach, beginning with an introduction to the topic, followed by a review of the state of the art and advancing to the methodology and system development description.

In the Section 1, the study’s context and motivation are presented, emphasizing the relevance of integrating technologies like IoT, data analysis, FIWARE, APIs, artificial intelligence, augmented reality and virtual reality in optimizing industrial maintenance. The research question, hypothesis and specific objectives of the study are also defined.

The Section 2 provides a comprehensive review of the literature, exploring the main concepts and technologies involved. This review positions the work within the current research context, identifying gaps and pointing out opportunities for future advancements.

The Section 3 describes the development process, starting with the requirements’ definition and including the data collection and analysis. This part covers prototype development; the integration of technologies such as IoT, FIWARE and AR/VR; the implementation of a REST API; and the use of an SQL database for information storage.

The Section 4 presents the architecture of the digital twin, detailing its various layers and components. These include the physical layer (sensors and devices), the digital layer (data structure and FIWARE), the cyber layer (AI functions), the interface layer (AR and VR applications) and the integration layer (using tools like Docker and a developed back office). It describes the development of the REST API and the database, explaining the structure and functionalities of the REST API, as well as the database used to store information about the machines, processes and maintenance history. The choice of a hybrid database, combining relational and graph models for more efficient data management, is highlighted.

The Use of FIWARE and Visualization with Grafana subsection addresses FIWARE’s implementation for real-time data management and integration with Grafana, enabling the intuitive and effective visualization of operational data to facilitate monitoring and decision-making. This section also includes the artificial intelligence for prediction and preventive action component, which explores the application of machine learning models, with a focus on linear regression, to predict critical trends such as temperature changes. Integration with FIWARE allows real-time analysis and the adoption of preventive actions based on predictions generated by the AI module. Finally, the development of augmented and virtual reality components is described. AR applications enable real-time visualizations and maintenance guidance, while VR applications create a virtual environment for training and the simulation of maintenance processes.

The Section 7 presents the outcomes of the system’s implementation. It describes the hardware configurations, such as the use of a Raspberry Pi and sensors, the analysis of the collected data and the operational gains achieved. The impact in terms of reducing downtime and increasing the predictive maintenance efficiency is evaluated.

The Future of the Project section discusses the potential to expand the system to integrate more machines and implement it in real industrial environments, enhance AI algorithms and explore the addition of new technologies like AR/VR glasses.

Finally, the Section 8 summarizes the main results of the study, reaffirming the importance of integrating the addressed technologies for industrial maintenance. Directions for future research are suggested, including the identified limitations and the practical implications of the obtained results.

## 2. State of the Art

This section addresses the key concepts and technologies underlying the development of a digital twin supported by augmented and virtual reality for predictive maintenance. This theoretical foundation is essential to understanding the context and significance of the project, as well as the technological basis supporting its implementation.

FIWARE, an open-source platform, facilitates the development of intelligent applications across various domains, such as Industry 4.0 and smart cities. By providing reusable components known as Generic Enablers, such as the Orion Context Broker, which manages real-time data [8], the platform has been applied in projects like smart buildings with extended digital twins (XDT). These projects integrate sensor data to monitor energy efficiency and occupant comfort while offering predictive simulations for optimized energy management [9].

The concept of a digital twin refers to creating accurate digital replicas of physical objects, systems or processes, continuously fed by real-world data. Widely adopted in industries such as manufacturing and healthcare, digital twins enable the simulation, monitoring and prediction of complex systems’ behaviors [10]. A practical case in the textile industry illustrates how digital twins monitor cutting machines and manual processes in real time, capturing data such as the temperature, vibration and operator movements to predict failures and enhance performance. Moreover, they help to identify employees prone to errors, facilitating tailored training interventions to boost efficiency [11].

To model processes associated with digital twins, Business Process Model and Notation (BPMN) is often used. BPMN provides a clear and precise visual representation using intuitive graphical symbols [12]. Its application in intelligent maintenance architectures [13] has proven effective in organizing workflows and identifying critical steps within connected and efficient Industry 4.0 environments [14].

The Internet of Things (IoT) complements digital twins by connecting physical devices that collect and share data over the Internet, ranging from simple sensors to complex systems. In the textile industry, IoT sensors monitor production processes and optimize machine maintenance in real time. One study demonstrated how IoT, combined with digital twins, enables predictive maintenance in wind turbines, improving asset management through sensor-based data [15,16]. However, IoT implementation requires robust infrastructure and appropriate interfaces for data acquisition, storage and access.

Predictive maintenance relies on data analysis and algorithms, such as linear regression, to anticipate equipment failures before they occur, minimizing downtime and costs [17]. For instance, in electric motors, sensors monitor variables such as vibration and the temperature, predicting the optimal intervention time and enhancing industrial equipment’s efficiency [18].

Augmented reality (AR) plays a crucial role by overlaying digital information onto the physical environment, improving the user interaction. In the industrial sector, AR provides real-time visual instructions, increasing the efficiency and accuracy of maintenance tasks [19]. According to [20], AR applications in industrial equipment maintenance have addressed issues such as prolonged wait times, lengthy training and project delays. AR has also been shown to enhance maintenance tasks’ precision and efficiency, reducing machine downtime [21].

Virtual reality (VR), on the other hand, creates simulated environments where users can interact with three-dimensional spaces. VR is particularly effective in operator training, reducing the costs and risks associated with traditional training methods. In an example described in [22], the system facilitates visual operations through interactive feedback and error identification during operator training. Furthermore, VR can be integrated with digital twins, allowing interaction with real-world environments through a virtual space. In [23], the authors reported the application of digital twins with VR to enhance the human–machine interaction in a smart manufacturing line, demonstrating improvements in task control and performance.

Finally, the integration of technologies such as digital twins, FIWARE, IoT, AR and predictive maintenance enables the creation of highly efficient systems. These systems monitor the real-time performance, provide visual support for complex tasks and predict maintenance needs based on usage patterns. This technological synergy is essential in implementing effective predictive maintenance, optimizing industrial operations and improving the overall process efficiency.

## 3. Methodology

The methodology applied in the development of the digital twin project, supported by augmented and virtual reality for predictive maintenance, encompasses several well-defined stages, the use of various tools and technologies and specific procedures for data collection and analysis. This section provides a detailed description of the project development process.

### 3.1. Specific Objectives

The specific objectives of this research include the development of a robust database designed to serve as a central repository for the critical operational data of machinery. This database aims to facilitate efficient process management, as well as the preventive maintenance of equipment. Subsequently, the implementation of Grafana is proposed for real-time data visualization, enabling operators to monitor the operational performance and quickly identify abnormal conditions that may arise.

Another key objective is the creation of an augmented reality (AR) application to present relevant operational data in real time, providing direct support for decision-making. Simultaneously, a virtual reality (VR) application will be developed, focusing on the visualization and manipulation of machinery within a digital environment. This application will use operational data to simulate maintenance procedures, facilitating operator training and preparation.

To connect these tools and ensure effective communication between the database and the AR and VR applications, an API will be implemented. This API will enable seamless, real-time interaction with operational data, offering users an integrated experience.

Additionally, this research aims to evaluate the effectiveness of an integrated system for subsequent adoption in real industrial environments. Through testing and validation, the goal is to determine the system’s ability to enhance predictive maintenance and operational safety, observing its direct impact on downtime reduction and the prevention of operational failures.

A future development objective includes the creation of a virtual training space for operators, where users can receive instructions on maintenance protocols in a safe environment, reinforcing the learning and practice of necessary procedures.

These objectives are interconnected and complementary, aiming to achieve significant advancements in equipment maintenance management and safety within industrial environments.

### 3.2. Development Process

The development of the project was carried out in sequential stages, each focusing on an essential component of the designed digital twin system. The process began with identifying the challenges and motivations that led to the creation of the project described here. Specific problems to be addressed were defined, ensuring a solid understanding of prior work in the field and establishing the value of the proposed solution.

Subsequently, the project needs were identified, and the functional and non-functional requirements of the system were established. During this phase, specific objectives were also defined for each module of the system, providing a clear and organized framework for the subsequent development stages.

During the initial prototype development phase, a basic digital twin prototype was implemented, employing sensors for data collection and transmission to the FIWARE platform. This created a foundational system for real-time monitoring and control. The initial configuration of FIWARE’s essential components, such as the Orion Context Broker, was carried out to manage real-time data and effectively integrate devices [8]. Additionally, a REST API was developed using Node.js [24] to facilitate communication between different system modules, enabling data exchange and the implementation of functionalities to manage complex entities such as machines and processes [25].

To ensure data robustness and organization, an SQL Database Management System was employed to store information about machines, processes, measurements and the maintenance history. This provided a solid structure for the management of the digital twin’s data [26]. Subsequently, Grafana was implemented to visualize the collected data, integrating seamlessly with FIWARE to monitor and display the data in an intuitive and real-time manner, thereby facilitating decision-making [8].

Augmented reality (AR) and virtual reality (VR) applications were also developed. The AR application enabled the real-time visualization of machine data and the creation of step-by-step sequences for predictive maintenance, optimizing operator interventions [27]. Meanwhile, the VR application facilitated the visualization of and interaction with machine data in a virtual environment, including simulated predictive maintenance sequences within the digital space, enabling operators to train in and practice these procedures.

The final stage involved rigorous testing and validation to ensure the system’s functionality and performance. Extensive tests were conducted, followed by multiple optimizations based on feedback from these tests, ensuring that the system met the proposed requirements and operated efficiently and cohesively.

### 3.3. Tools and Technologies

The development of the project utilized a variety of carefully selected software and hardware tools to ensure the system’s efficiency and effectiveness. This section details the main tools used, categorized according to their roles in the proposed system.

#### 3.3.1. FIWARE and Data Management

FIWARE played a central role in the developed system, serving as the primary data management platform. Its modular, scalable and open-source architecture make it an ideal choice for industrial applications requiring the efficient integration of IoT devices and analytical services. Among the most critical components used was the Orion Context Broker, responsible for managing real-time contextual information [8]. This module collects, stores and makes available data from various sources, such as IoT sensors, monitoring systems and analytics modules.

In the developed system, the Orion Context Broker enabled the collection and organization of sensor data, such as the temperature, vibration and machine states, ensuring that all information was accessible and centralized. This approach ensured interoperability between different system components while facilitating a continuous data flow, even in dynamic and distributed environments. Compared to other data management platforms, such as AWS IoT Core and Azure IoT Hub, FIWARE stood out due to its open-source nature, reducing the implementation costs without compromising its flexibility or integration capabilities.

To complement FIWARE’s functionality, a REST API was developed using Node.js. This API facilitated communication between system modules, allowing FIWARE-managed data to be efficiently accessed and manipulated by integrated services and technologies. Additionally, the REST API provided a simplified interface for external interactions, enabling aspects such as dashboard monitoring and data analysis algorithms to utilize real-time information.

The integration of the Orion Context Broker with the REST API brought significant advantages, including a consistent data flow and scalability to support new features. For example, in predictive maintenance scenarios where IoT sensors report information asynchronously, FIWARE ensures that data are stored and made available for analysis without delays or losses. This is critical for the early detection of anomalies and in executing preventive actions based on reliable information.

Another crucial benefit is FIWARE’s adherence to open protocols like NGSI-LD, which allows seamless integration with other compatible tools and services. This compatibility not only facilitated system development but also ensured adaptability for future expansions and the incorporation of new technologies. This characteristic is especially relevant in industrial contexts, where the monitoring and analysis requirements can evolve rapidly.

Together, FIWARE and the developed REST API formed the backbone of the data management system, providing a robust foundation for the integration of various technologies. The choice of FIWARE was strategic, enabling the creation of a modular and efficient system aligned with the needs of predictive maintenance while being prepared to support technological advancements in real industrial scenarios.

#### 3.3.2. Machine Learning Models

In the domain of data analysis and failure prediction, a linear regression model was implemented using Python 3.12.0 and TensorFlow 2.17.0. This model was chosen for its simplicity and efficiency in initial prototyping contexts [28,29]. Although more advanced models exist, such as deep neural networks [30], random forest [31], and support vector machines, linear regression was selected to demonstrate the feasibility of integrating technologies and to highlight that simple approaches can produce relevant results.

This approach was motivated by two main factors. First, linear regression is widely understood and easy to implement, facilitating its initial integration with other technologies, such as FIWARE (FIWARE Foundation, Berlin, Germany). Second, the project’s goal was not to achieve the maximum predictive model performance but to validate the proposed architecture and its ability to operate efficiently in real time. Future iterations will explore more sophisticated models to enhance the predictive accuracy while maintaining the established technological foundation.

#### 3.3.3. Data Visualization with Grafana

Grafana played a pivotal role in data visualization, functioning as a powerful interface to present real-time information in a clear and intuitive manner. This software was directly integrated with FIWARE, using QuantumLeap as an intermediary to convert contextual data managed by the Orion Context Broker into time-series data. This conversion enabled Grafana to create dynamic and interactive dashboards, offering a comprehensive view of the system’s status [8].

One of Grafana’s main advantages lies in its flexibility. This platform allows users to customize the dashboards to meet specific needs, providing diverse visualization options such as line charts, tables and visual alerts. In the developed system, this functionality is fundamental for continuous monitoring and early anomaly detection, as operators can track critical indicators like the temperature, vibration and overall machine performance in real time.

Another highlight is Grafana’s capability to integrate with various data sources. In the system, besides FIWARE, the platform was configured to support future expansions, such as incorporating data from other industrial monitoring tools. This compatibility ensures that the system remains scalable and adaptable to the growing needs of industrial environments.

Grafana’s intuitive interface was a key factor in its selection. Unlike other commercial solutions, such as Tableau or Power BI, Grafana combines the robustness of an advanced tool with ease of use, making it accessible to both experienced technicians and operators with limited technical training. This accessibility was crucial in ensuring that real-time monitoring was effective and seamlessly integrated into users’ daily workflows.

#### 3.3.4. Augmented Reality Technologies

The development of augmented reality (AR) functionalities in the system was supported by the use of the Vuforia platform, one of the most advanced solutions available for AR applications. Vuforia is renowned for its capabilities in image and object recognition, enabling the creation of interactive and immersive experiences that enhance user perception [32]. In the context of this project, the platform was essential for transforming abstract data into accessible visual representations, facilitating interaction and understanding for operators in industrial settings.

Vuforia was selected for its robustness and versatility, allowing for the integration of features such as marker recognition (e.g., QR codes or specific patterns) and the real-time tracking of three-dimensional objects. For instance, in industrial scenarios, Vuforia enables the overlay of digital information, such as maintenance instructions or operational alerts, directly onto physical machines or equipment. This functionality is critical in improving the operational efficiency by reducing the time required to locate and interpret critical information.

The integration of Vuforia was carried out with Unity, one of the most widely used game engines for the development of interactive applications and immersive experiences. Unity provided a flexible development environment, rich in resources and compatible with various AR devices, such as smartphones, tablets and smart glasses [33]. This flexibility was essential in ensuring that the developed solutions could be adapted to different contexts and requirements, ranging from mobile applications to advanced systems based on specific hardware.

The Vuforia SDK, combined with Unity’s extended reality (XR) capabilities, allowed the creation of highly customizable AR interfaces tailored to industrial scenarios. Using this suite of tools, several functionalities were developed, including the following.

- Real-Time Data Overlay: Information such as sensor readings or maintenance instructions was projected directly onto equipment, aiding operators in decision-making.
- Visual Guides for Critical Operations: Through arrows, text or animations overlaid on the physical environment, users were guided in real time to perform specific maintenance or repair tasks.
- Object and Pattern Recognition: The platform identified and tracked physical objects, even in dynamic environments, ensuring precision in the presentation of contextual information.

This combination of technologies brought significant benefits to the system. Augmented reality enhanced operators’ safety and efficiency by reducing human error through the visual and accessible presentation of critical information. Additionally, the flexibility of Unity and Vuforia ensured that the system could be easily scaled or modified to meet the needs of various industries and operational scenarios.

The strategic choice of Vuforia and Unity also ensured compatibility with future technologies. Both platforms are continuously evolving, incorporating new features and maintaining support for emerging devices such as smart glasses and depth cameras. This adaptability guarantees that the developed system will remain relevant and flexible, providing a solid foundation for future expansions and improvements.

#### 3.3.5. Virtual Reality with Meta Quest 2

To test and implement the virtual reality (VR) applications, the Meta Quest 2 headset was used, being one of the most versatile devices available on the market. These headsets were chosen for their ability to provide a fully immersive experience, combining graphical quality, comfort and an intuitive user interface. In the context of this project, the Meta Quest 2 played a crucial role by enabling the creation and exploration of virtual environments replicating complex industrial scenarios [34].

The use of Meta Quest 2 was fundamental in assessing the impact of VR in the context of predictive maintenance. These devices allowed realistic simulations of industrial environments, offering users the opportunity to interact with virtual machines and equipment under conditions close to real-world scenarios.

One of the main benefits of the Meta Quest 2 is its integration with development platforms such as Unity, which facilitated the creation of highly detailed three-dimensional environments. Using Unity’s extended reality (XR) features, it was possible to design interactive scenarios where users could achieve the following.

- Explore Machines in Detail: Visualize internal components of industrial equipment through interactive 3D models.
- Simulate Maintenance Procedures: Perform repair or adjustment steps on virtual equipment with real-time visual and audio feedback.
- Respond to Emergency Scenarios: Participate in simulations of failures or anomalies, learning to respond quickly in critical situations.

In addition to its technical capabilities, the Meta Quest 2 stood out for its portability and ease of setup. Unlike other VR devices that require powerful computers and complex installations, the Meta Quest 2 operates independently, significantly reducing the costs and logistical efforts. This simplicity was essential in integrating the technology into the project, enabling quick and efficient testing.

#### 3.3.6. Context and Perspectives

The chosen tools and technologies were carefully integrated to form a cohesive and functional system, where each component played a specific role, complementing the others in creating a viable prototype. The deliberate selection of emerging technologies, such as FIWARE, Grafana, Vuforia, Unity and the Meta Quest 2, was guided by their ability to meet the specific requirements of predictive maintenance in modern industrial scenarios. This integrated approach demonstrates not only the effectiveness of these technologies individually but also their potential when combined, creating an innovative and robust solution.

One of the main strengths of the developed system lies in its flexibility and adaptability. By opting for modular and scalable technologies such as FIWARE and Grafana, it was possible to build an architecture that not only meets the current needs but is also prepared to incorporate future improvements and expansions. For example, integrating new IoT sensors, more sophisticated predictive analysis algorithms or advanced user interfaces can be achieved seamlessly, leveraging the existing structure.

The system demonstrates that simple approaches, such as using linear regression for predictive analysis, can be effective when integrated into a well-designed architecture. This balance between simplicity and technological innovation was fundamental in validating the prototype’s feasibility and ensuring that the proposed solutions were accessible and replicable in different industrial contexts. The choice of open-source tools, such as FIWARE and Unity, reinforces this vision by providing a cost-effective alternative for companies seeking to adopt advanced technologies without incurring prohibitive costs.

From a practical perspective, the prototype not only validated the system’s functionality but also highlighted areas with potential for improvement and evolution. Augmented reality and virtual reality, for instance, proved to be powerful tools in transforming human–machine interaction, increasing the efficiency and safety in maintenance processes. However, future iterations may explore more advanced devices, such as mixed-reality glasses, or integrate haptic feedback systems to make interactions even more realistic and immersive.

The future prospects are equally promising. The implementation of more advanced artificial intelligence algorithms, such as convolutional neural networks or ensemble techniques, will allow for improved prediction accuracy and expanded application scope. Furthermore, combining historical and real-time data could open new possibilities for the creation of hybrid predictive models, optimizing the responsiveness to critical events.

### 3.4. Data Collection

The procedures for data collection and analysis were carefully planned to ensure the accuracy and relevance of the information.

- Sensor Installation: Temperature and humidity sensors were installed on the machine to collect real-time data.
- Data Transmission to FIWARE: The HTTP protocol was used to send sensor data to the Orion Context Broker, where they were managed in real time [8].
- Historical Data Storage: Historical data were stored using QuantumLeap and CrateDB, allowing for detailed analyses and future predictions [8].
- Data Analysis: Machine Learning algorithms were applied to the previously stored historical data to identify patterns and predict maintenance needs, thereby improving the system’s operational efficiency [26].

## 4. System Architecture and Development

The architecture of the digital twin system, supported by augmented and virtual reality for predictive maintenance, consists of multiple layers and components designed to ensure efficiency and effectiveness. A detailed description of each layer and its respective components is provided below.

### 4.1. Overview

**Figure 1:**
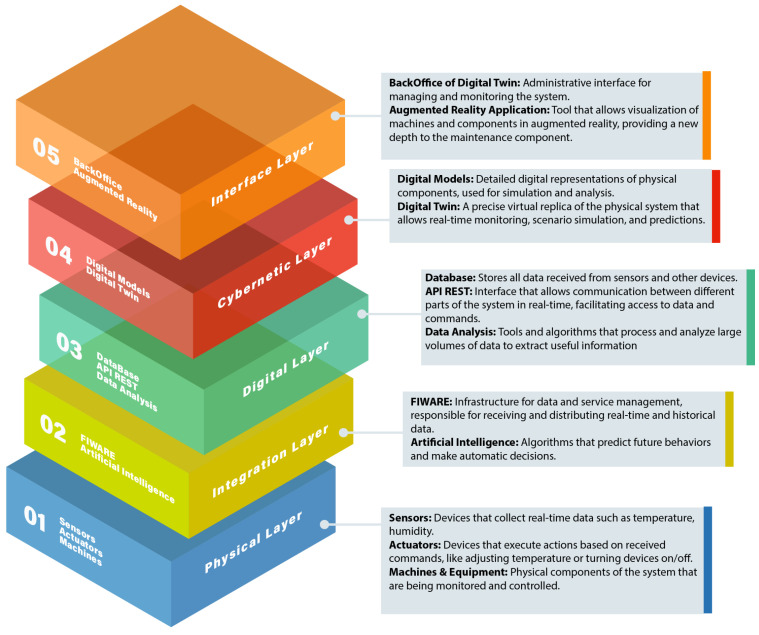
Layered system architecture.

The system architecture is structured into five layers, each playing a specific role in the operation of the digital twin (Figure 1). These layers include the physical layer, digital layer, cyber layer, interface layer and integration layer. Each of these layers interacts to collect, process, analyze and present real-time data, facilitating predictive maintenance and the efficient management of industrial processes.

#### 4.1.1. Physical Layer

The physical layer comprises physical devices, including sensors, responsible for collecting data from the physical environment. One example is the DHT11 sensor [35], which monitors the temperature and humidity variables. This sensor is connected to a Raspberry Pi computing device, which serves as the primary component of this layer. These physical devices play an essential role by enabling the collection of information from machines and their surrounding environment, transmitting the collected data to the digital layer via communication protocols such as HTTP. The Raspberry Pi acts as an intermediary, receiving sensor data and transmitting them to the FIWARE platform, ensuring seamless integration between physical data and the digital system.

#### 4.1.2. Integration Layer

System integration is facilitated through the use of Docker containers, which ensure consistency across development and production environments. Docker is employed to package applications and their dependencies, ensuring that they run uniformly across different setups. Notably, the support back office plays a crucial role by offering functionalities for data visualization, notifications and alerts, maintenance management and user administration. This back office also enables the expansion of artificial intelligence capabilities by integrating predictive models and performance analyses. Communication between the system’s various components is managed by the Docker network, providing isolation and interaction between containers, thus simplifying application development, testing and deployment.

#### 4.1.3. Digital Layer

The digital layer is responsible for organizing and storing the data collected by the physical layer. An SQL database is utilized to store detailed information about machines, processes, measurements and the maintenance history. Tools such as QuantumLeap are used to store historical data, enabling more in-depth analyses and data predictions. The FIWARE platform, particularly the Orion Context Broker, is employed to manage real-time data, facilitating communication between IoT devices and applications. The Orion Context Broker specifically supports real-time visualization and interaction with the operational data of the machines.

#### 4.1.4. Cyber Layer

The cyber layer focuses on data analysis and prediction, using machine learning algorithms such as linear regression. This layer is critical in monitoring machine performance, predicting failures and optimizing operations based on real-time and historical data. Machine learning algorithms identify patterns in the collected data, enabling the prediction of potential failures or maintenance needs. This allows proactive actions to be implemented, aiming to prevent unexpected machine downtime and ensuring greater operational efficiency.

#### 4.1.5. Interface Layer

The interface layer is dedicated to the interaction between the user and the system. The user interface is designed to provide the real-time visualization of the data collected from the machines. The augmented reality (AR) application allows operators to view data directly in the physical environment and receive detailed maintenance instructions overlaid on the real-world scene. Meanwhile, the virtual reality (VR) application offers an immersive environment for practical training, enabling the simulation of maintenance tasks. Both approaches significantly enhance the efficiency and accuracy of maintenance operations, promoting a more intuitive and functional user experience.

### 4.2. System Architecture Overview

The architecture of the system developed for the digital twin project, supported by augmented and virtual reality for predictive maintenance, comprises multiple layers of hardware and software components, which collectively ensure the system’s efficiency and effectiveness.

### 4.3. Application Process Diagram

**Figure 2:**
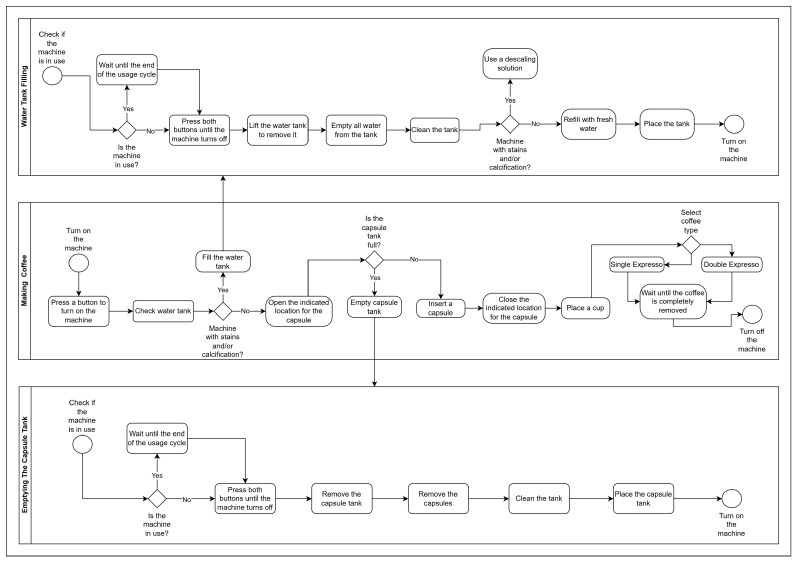
BPMN diagram representing the maintenance processes of a coffee machine.

The process diagram (BPMN) developed (Figure 2) provides a comprehensive overview of the functionality of the augmented reality application for the predictive maintenance of coffee machines. It represents the various steps and decisions involved in the operation and maintenance process in detail. By analyzing this diagram, users can understand how the application supports them in executing specific actions, from preparing a coffee to cleaning and maintaining the water reservoir. The diagram facilitates an understanding of each step, ensuring that operators correctly follow the procedures to keep the equipment in optimal working condition.

The diagram is organized into three main sections.

- Emptying the Water Reservoir: This initial process describes the steps to check and empty the machine’s water reservoir. The process begins with verifying the machine’s state: in use or idle. If the machine is in use, it is necessary to wait until the cycle finishes before proceeding. When the machine is idle, the operator can turn it off, lift the reservoir and empty the water. If signs of calcification are present, the diagram provides guidance on how to use a descaling solution, wash the reservoir and finally refill it with fresh water before placing it back in the machine. This process ensures the removal of accumulated impurities, promoting the equipment’s longevity and coffee quality.
- Preparing a Coffee: This process focuses on coffee preparation, from turning on the machine to selecting the desired coffee type (short or long). Initially, the user turns on the machine and checks whether the water reservoir needs refilling. If not, the next step is to check if the capsule container is full. If it is full, the container should be emptied; otherwise, the user can insert a new capsule. After the user closes the capsule compartment, they place a cup and select the desired coffee type. Once the machine provides the chosen coffee, it can be turned off. This process connects directly with the other two processes when refilling the water reservoir and/or emptying the capsule container.
- Emptying the Capsule Container: The final section of the diagram illustrates the procedure for emptying the capsule container, essential in keeping the machine in good operational condition. The process begins with verifying that the machine is not in use. If it is in use, the operator waits for the cycle to finish. Once the machine is free, it can be turned off, the capsule container removed, the capsules disposed of and the necessary cleaning performed. Afterward, the container is placed back in the machine, which can then be turned on again, ensuring that it is ready for a new coffee preparation cycle.

Each of these sections of the diagram allows the user to perform predictive maintenance procedures in a clear and organized manner, minimizing the risk of operational errors and promoting the machine’s preservation.

### 4.4. Database Development

**Figure 3:**
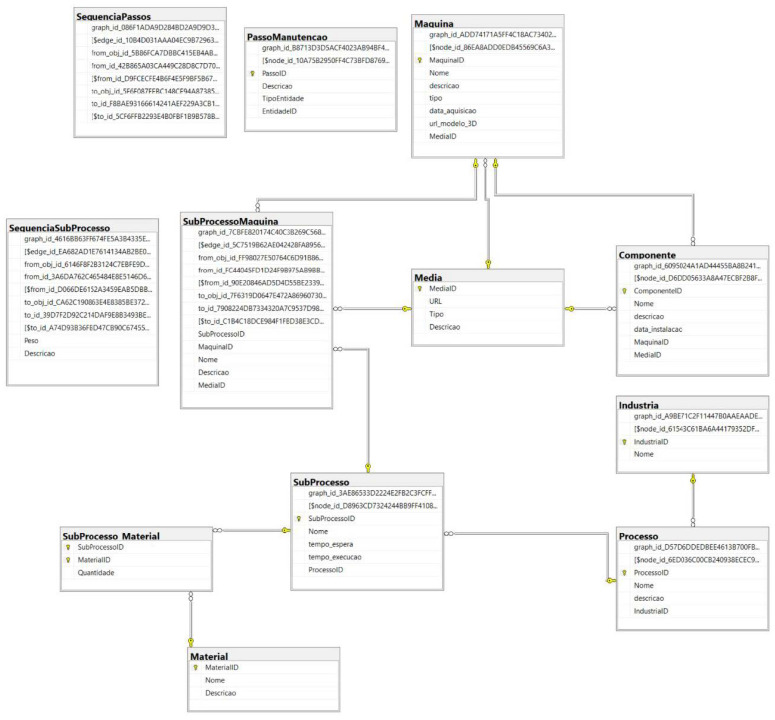
System database structure.

The database developed for the project uses a hybrid SQL system, combining relational and graph structures. The relational part stores transactional data about machines and processes, while graphs are used to represent and analyze maintenance sequences and dependencies, as illustrated in Figure 3. This hybrid approach provides more intuitive and efficient data management, especially for complex queries involving relationships between elements.

Among the main advantages of this approach is the intuitive representation offered by the graphs, which map the connections clearly, making them ideal for complex processes such as maintenance. Additionally, the analysis of sequences or dependencies is significantly faster in graphs than in relational structures, which require more complex operations for similar queries. The system’s flexibility allows adaptations to new requirements with minimal changes, while the advanced analytical capabilities facilitate the identification of patterns and bottlenecks in processes.

A practical example of this hybrid approach is its application in a textile factory, where the database is used to manage production and maintenance. The production structure is organized into tables containing information about industries, processes, subprocesses and machines, while graphs connect subprocesses to optimize production flows and reduce failures. For maintenance, the tables offer data about machines, components and necessary steps, while graphs help to create logical sequences that ensure that tasks are executed correctly, preventing unplanned downtime. This integration of relational and graph data proves essential in meeting complex production and maintenance demands efficiently and scalably.

The data model uses various interconnected tables to manage an industrial system. The Industry table is related to the Process table, which organizes the main processes by industry. Each process is detailed in the SubProcess table, which describes specific steps and also relates to the Material table through the SubProcess Material table, indicating the materials used in each step. TheSubProcessSequence table defines the execution order between subprocesses.

In terms of maintenance, the Machine table details the equipment used, while the Component table describes the individual parts of these machines. The MaintenanceStep and StepSequence tables organize the steps and the order of maintenance, linking to the machines and components. The Media table associates multimedia information with machines, components and other elements, enriching the system’s documentation.

### 4.5. Development of the REST API

**Figure 4:**
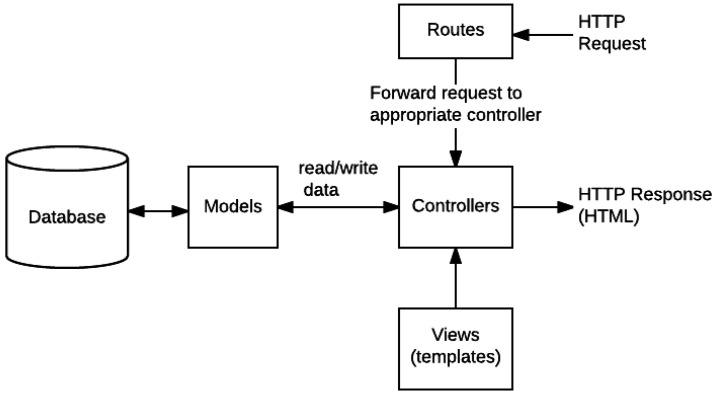
REST API structure.

To facilitate the management of the digital twin, a REST API was developed, composed of several components that enable efficient and standardized communication and data manipulation between the client and server. The structure includes models, which define the data structure and ensure its integrity; controllers, responsible for processing requests and executing business logic; and routes, which map HTTP requests to specific controllers, organizing the API into clear endpoints [36]. See Figure 4.

The API handles various complex entities, such as machines and processes, through CRUD operations. It also facilitates communication between system modules, enabling real-time data visualization and manipulation [24].

### 4.6. FIWARE Utilization and Data Visualization with Grafana

**Figure 5:**
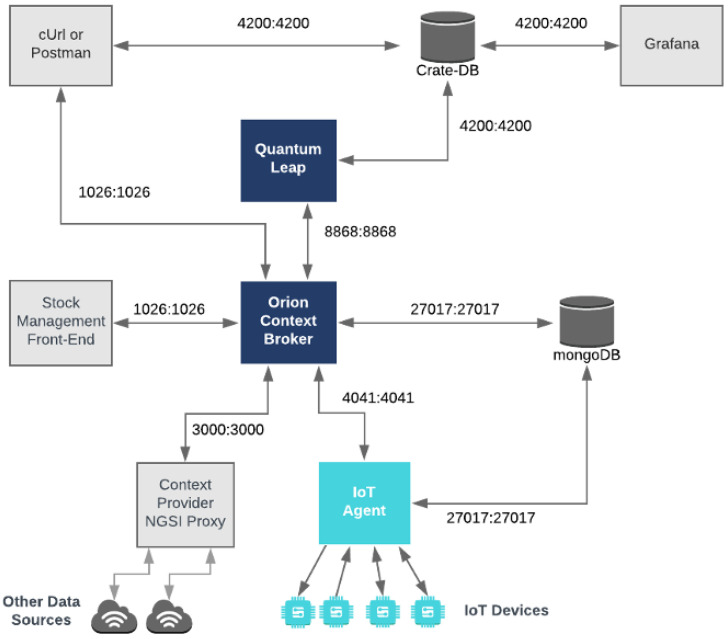
FIWARE structure within the system.

FIWARE is an open-source platform that provides APIs for the development of intelligent applications and management of real-time contextual data. The Orion Context Broker handles real-time contextual data, facilitating communication between sensors and services, while QuantumLeap stores historical data for predictive analyses (Figure 5).

**Figure 6:**
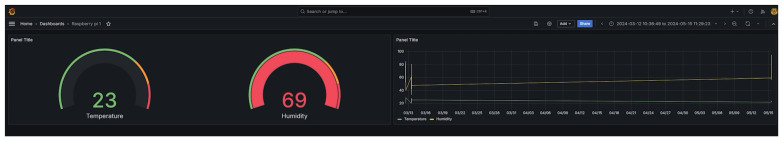
Example of a dashboard using Grafana.

Grafana is a data visualization platform that allows the creation of interactive dashboards. Grafana integrates with QuantumLeap and the Orion Context Broker, offering a comprehensive view of the system’s state, as shown in Figure 6, enabling the visualization of real-time and historical data collected by FIWARE [37].

### 4.7. Artificial Intelligence for Prediction and Preventive Action

The developed system uses a simple artificial intelligence algorithm to assist in predicting the values of variables provided by the sensors. In this case, the sensor used collects, in real time, the temperature and humidity values of a specific physical space where a machine is located. These values are then sent to the AI module, which analyzes, processes and calculates predictions based on the sequence of received data values.

## 5. Linear Regression Model

To estimate temperature predictions based on historical data, a linear regression model was implemented using the sklearn.linear_model library. This model is suitable for data with approximately linear behavior, as it identifies the relationship between an independent variable (time) and a dependent variable (temperature).

### 5.1. Training and Testing Process

The process begins with the collection of historical data, which are divided into two sets: training and testing. The training set is used to fit the model and determine regression coefficients, while the testing set evaluates the model’s predictive capabilities on new data. During training, the model fits a straight line to the data by minimizing the error between the predictions and observed values through the least squares method. This method solves the following equation to find the optimal values of $\theta_{1}$ (intercept) and $\theta_{0}$ (slope):

$$y=\theta_{1}+\theta_{0}x$$

The slope ($\theta_{0}$) represents the rate of temperature change over time, while the intercept ($\theta_{1}$) indicates the initial temperature value when x=0.

### 5.2. Workflow Description

**Figure 7:**
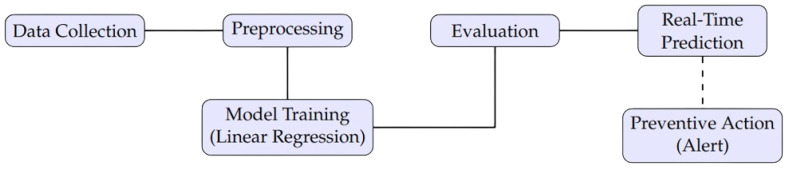
Process workflow from data collection to preventive actions.

Figure 7 presents the workflow of the process, from data collection to the application of preventive actions. The diagram illustrates the main steps.

- Data Collection: Sensors collect the temperature and other operational parameters in real time.
- Preprocessing: Data are processed to remove outliers and normalize the values.
- Model Training: Historical data are used to fit the regression model.
- Evaluation: The model is tested to verify the prediction accuracy.
- Real-Time Prediction: The model is integrated into a system that provides temperature forecasts.
- Preventive Actions: Alerts are generated for anomalous conditions, triggering maintenance actions.

### 5.3. Prediction Calculations

**Figure 8:**
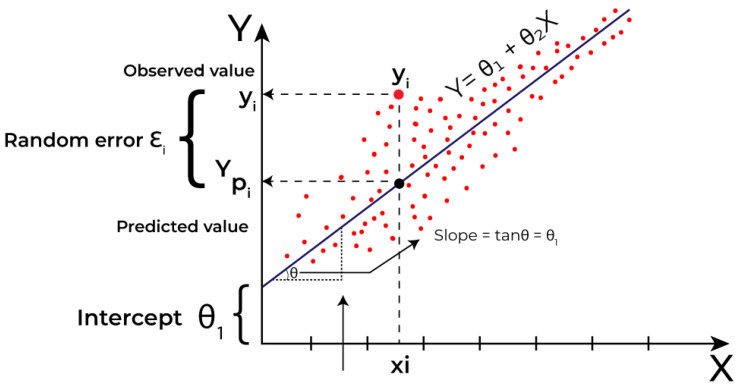
Calculation of future temperature predictions.

Figure 8 illustrates the calculation of future temperature predictions, highlighting the following.

- ***y***: Observed value (red points), representing the actual temperature recorded.
- **$y_{p}$**: Predicted value from the linear regression model, calculated using the formula.
- **$\epsilon_{i}$**: Residual error, which is the difference between the observed value (*y*) and the predicted value ($y_{p}$).
- **$\theta_{1}$**: Intercept, representing the initial temperature value.
- **$\theta_{0}$**: Slope of the line, indicating the relationship between the time and temperature.

This adjustment minimizes the total error ($\epsilon_{i}$) and provides reliable predictions based on historical patterns.

### 5.4. Integration and Practical Applications

The integration of the model with FIWARE enables the collection, processing and visualization of real-time data. Using QuantumLeap for historical storage and Grafana for dynamic visualization, the system is capable of the following.

- Monitoring Anomalies: Identifying abnormal trends, such as sudden temperature increases, which may indicate imminent failures.
- Preventing Failures: Generating automatic alerts for corrective actions, reducing machine downtime.
- Optimizing Maintenance: Planning preventive interventions based on concrete data, avoiding reactive maintenance and improving the operational efficiency.

For instance, in a textile factory, the system can predict motor overheating, allowing the technical team to intervene before damage occurs. Similarly, in refrigeration environments, temperature deviations can be quickly corrected to preserve the quality of the final product.

Continuous visualization and real-time predictions make the system an essential tool for predictive maintenance, aligned with the principles of Industry 4.0.

The integration of the model with FIWARE enables the collection, processing and visualization of real-time data. The data collection function retrieves data from QuantumLeap, prepares them in a DataFrame and uses the model represented in Figure 8 to predict future temperatures. The visualization is dynamically updated, providing an interactive and intuitive graphical interface for real-time monitoring [38,39].

## 6. Interactive Applications

### 6.1. Augmented Reality Application

An augmented reality application was developed as part of a simulation scenario for the maintenance of a coffee machine. The application provides essential functionalities to assist an operator in the machine maintenance process. Scripts were programmed in C# to enable communication with the developed APIs, gathering all relevant data within the application. Using the Vuforia SDK within a Unity development environment, it is possible to detect a physical object based on a pre-designed 3D digital object created in Blender and stored in a Vuforia database. Once the physical machine object is detected by the camera of a tablet or smartphone, the digital model of the machine is overlaid in real time.

**Figure 9:**
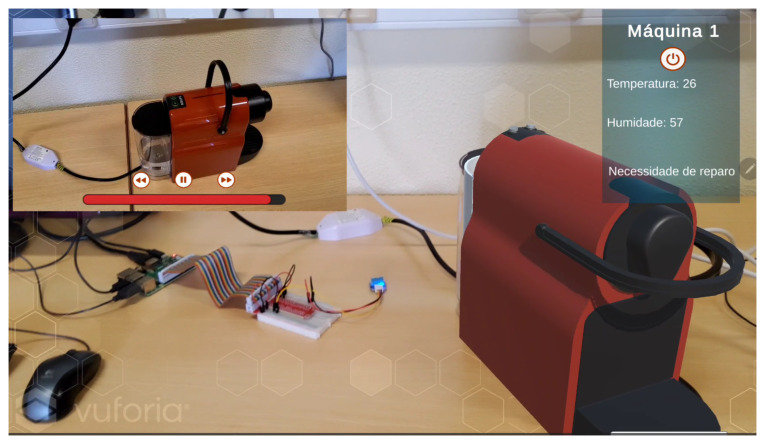
Augmented reality application.

Figure 9 shows the user interface of the application. This interface includes two key areas:

- In the top-left corner of the screen, multimedia content is displayed for the various steps involved in performing machine maintenance, including controls for play/pause functionality in a video;
- In the top-right corner of the screen, information obtained from physical sensors (temperature and humidity) is displayed, along with additional critical information.

If any value read from the physical sensors is deemed hazardous, the user can remotely turn off the machine using a virtual button. This triggers a physical switch implemented within the coffee machine, cutting off its power supply.

The interactive maintenance step sequence stands out as a valuable resource for the training of technicians and operators, offering structured and practical learning. This is reinforced by explanatory multimedia content and 3D animations that visually detail each step of the process.

### 6.2. Virtual Reality Application

**Figure 10:**
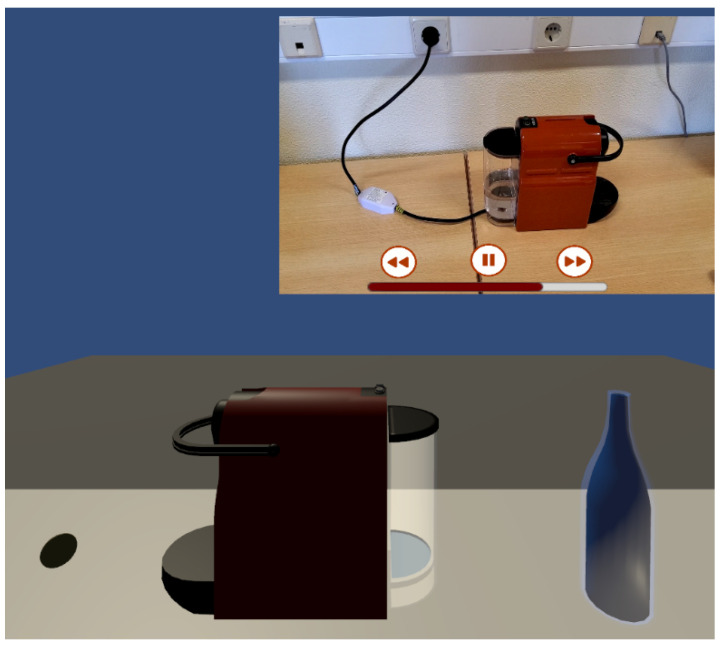
Interaction with objects in a virtual environment.

A virtual reality (VR) application was also developed to simulate maintenance training scenarios within a virtual environment. Using scripts written in C#, relevant data are integrated and presented in a virtual space created in Unity with the XR Interaction Toolkit. This space allows the operator to view and interact with digital models of the machine and all components required for maintenance operations (Figure 10).

The machine model used in the application was also developed in Blender and represents an advanced version of the model designed for the AR application. It allows for subdivision into the necessary elements for operation and maintenance. The VR application provides an accurate representation of the virtual space, enabling interaction with virtual objects, detailed real-time data visualization and an interactive maintenance step sequence similar to the AR application.

Additionally, for the training of technicians, the VR application offers access to personalized multimedia content, such as demonstrative and interactive videos of the required steps and sensor information displayed in the operator’s field of view. This immersive environment allows the operator to follow instructions while interacting with the available 3D digital models in the application.

The interactive models composing the machine include the following:

- The water tank, which the user can fill through simultaneous interaction with a water bottle;
- The lever, which can be rotated to allow the insertion of a capsule;
- The cup, which can be placed in the machine;
- The buttons, which are used to select different types of coffee;
- Finally, the capsule container, which can be removed for emptying.

## 7. Results

As a result, an initial validation test of the developed system was conducted to evaluate its potential effectiveness in predictive maintenance and its future evolution into a real system in an industrial environment. For this purpose, the virtual reality application was tested with users, and their feedback was collected using the *System Usability Scale* (SUS) questionnaire [40]. The SUS is widely used to measure the usability of systems through a 10-item questionnaire scored on a 5-point Likert scale, ranging from “Strongly Disagree” to “Strongly Agree”.

The augmented reality application was tested by 15 participants aged between 22 and 28, who subsequently completed the SUS questionnaire. The responses provided important insights into the application’s usability. From the analysis of the questionnaire responses, an average score of 78.8 was obtained, indicating above-average usability, as a score of 68 or higher is considered acceptable.

### 7.1. SUS Questionnaire Items

Below are the 10 items that compose the SUS questionnaire, along with a brief explanation of their purpose.

1. Q1: I think that I would use this application frequently.
2. Q2: I found the application unnecessarily complex.
3. Q3: I thought the application was easy to use.
4. Q4: I think I would need the support of a technical person to be able to use this application.
5. Q5: I found the various functions in this application were well integrated.
6. Q6: I thought there was too much inconsistency in this application.
7. Q7: I imagine that most people would learn to use this application very quickly.
8. Q8: I found the application very cumbersome to use.
9. Q9: I felt very confident using the application.
10. Q10: I needed to learn a lot of things before I could get going with this application.

### 7.2. Detailed Analysis of the Results

**Figure 11:**
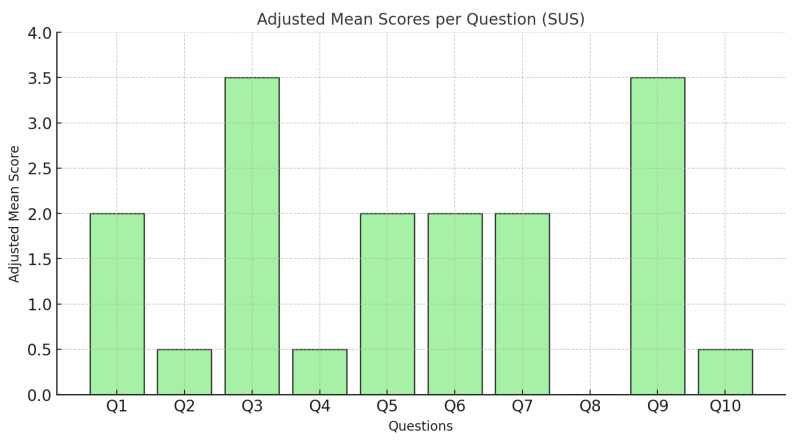
Average adjusted scores per question (SUS).

Figure 11 shows the adjusted scores per question. A detailed analysis is provided below.

- Highest-scoring questions (Q3, Q9): These questions indicate that participants found the application easy to use (Q3) and felt confident while using it (Q9). These results highlight the interface’s effectiveness in supporting the tasks performed.
- Moderately scoring questions (Q5, Q7): Questions related to the integration of functionalities (Q5) and ease of learning (Q7) received good evaluations but suggest room for improvement. Qualitative feedback indicated that adding tutorials could further facilitate initial use.
- Lowest-scoring questions (Q2, Q4): Question Q4, regarding the need for technical assistance, received lower scores. This suggests that a small portion of users experienced difficulties in performing certain tasks. Conversely, the low score for Q2 reinforces that the application is not perceived as complex.
- Question Q8 (application inconsistency): Most participants disagreed that the application was inconsistent. This score reflects the system’s robustness in providing a uniform user experience.

### 7.3. Conclusions from Results

The results suggest that, overall, the application was well received by the participants, demonstrating above-average usability. Questions such as Q3 and Q9 indicate that the application is intuitive and inspires confidence in users. However, areas such as the need for technical assistance (Q4) and the integration of functionalities (Q5) could be improved. It is recommended to include detailed tutorials and simplify the interface to better accommodate less experienced users.

The detailed analysis of the questions provides valuable insights for future iterations of the system, allowing for enhancements to the overall user experience and ensuring greater efficiency in industrial environments.

## 8. Conclusions

This study concludes by summarizing the key points addressed throughout the article, highlighting that the integration of emerging technologies, such as a FIWARE-based digital twin, augmented reality (AR) and virtual reality (VR), has demonstrated potential to significantly support operators in industrial maintenance processes. The developed system shows the potential of these technologies to enhance machine efficiency and operational safety by enabling precise monitoring, early anomaly detection and more effective and secure maintenance execution. Integrating these technologies enhances the ability to predict failures and deliver detailed visual instructions, reducing machine downtime and improving the operational efficiency.

The relevance of the results lies in validating the initial hypothesis: the combination of IoT, data analysis and AR/VR can create an effective predictive maintenance system. This system demonstrated the potential to improve operational safety by alerting operators to anomalous conditions and optimizing maintenance processes through an intuitive and visually rich interface. Furthermore, the practical benefits of the system have significant implications for the industry, showing how emerging technologies can be integrated to address real-world problems.

Although the present study demonstrated promising results, it is important to note that a comparative analysis with advanced methods has not yet been conducted. This decision was due to the exploratory nature of the work, whose primary objective was to validate the technological integration and test the feasibility of a functional prototype in industrial contexts. The choice of linear regression, despite its simplicity, was deliberate to prioritize simplicity, efficiency and ease of implementation in a prototyping environment. More sophisticated models, such as deep neural networks or random forest, will be explored in future iterations, allowing for a more detailed analysis of the system’s predictive effectiveness compared to other approaches. However, the results obtained show that even simple approaches can be effective when integrated into a robust technological architecture.

The initial results are promising, and, to further validate the outcomes, it will be necessary to expand the testing to different types of equipment and varied industrial environments. Additionally, the AI component can be developed with the integration of more sophisticated models and larger datasets for training, improving the prediction accuracy. The exploration of new augmented and virtual reality technologies also holds significant development potential, enhancing the user interface and interaction with the system.

Given the promising results obtained, this project’s future involves broadening the reach of the developed solutions and validating their application in real industrial environments. Key objectives include incorporating industrial machinery into the system, enabling broader and more varied data collection to provide a more comprehensive view of industrial operations. Transitioning to real industrial environments will be essential to validate the system’s effectiveness in practical scenarios, refining and improving the solution based on observed challenges and opportunities. Integrating these improvements will help to establish this system as a robust and innovative solution for predictive maintenance in Industry 4.0.

## Data Availability

Data are contained within the article.
